# Shunting outcomes in post-hemorrhagic hydrocephalus: A protocol for systematic review and meta-analysis

**DOI:** 10.1097/MD.0000000000021640

**Published:** 2020-08-07

**Authors:** Tong Sun, Junwen Guan, Chao You, Jingguo Yang, Xuepei Li, Yikai Yuan, Yicheng Zhou

**Affiliations:** a Department of Neurosurgery; b West China Brain Research Center, West China Hospital; c Health Ministry Key Laboratory of Chronobiology, West China Medical Center, Sichuan University, Chengdu, Sichuan, PR China.

**Keywords:** post-hemorrhagic hydrocephalus, ventriculoperitoneal shunt, lumboperitoneal shunt, treatment outcomes

## Abstract

**Background:**

The best treatment option for patients with post-hemorrhagic hydrocephalus (PHH) remains controversial. The objective of the current meta-analysis is to systematically evaluate the long-term outcomes of patients with PHH treated by ventriculoperitoneal shunt (VPS) and lumboperitoneal shunt (LPS).

**Methods:**

We search literatures through PubMed, Web of Science, Embase, Cochrane Library, China National Knowledge Infrastructure (CNKI), the Chinese Science and Technology Periodical Database (VIP) and Wan fang databases, and Chinese Biomedical Literature Database (CBM) from its beginning to June 15, 2020. Randomized controlled trials (RCTs) and non-RCTs in English or Chinese studies will be considered. The primary outcome is the rate of shunt failure after shunt implantation

The secondary outcome is the rate of complications that are associated with shunt surgery.

**Results and conclusion:**

The study will compare the 2 types of shunt surgery in the treatment of PHH, providing evidence for the treatment option for the patients with PHH.

**Study registration number:**

The study is priorly registered through International Platform of Registered Systematic Review and Meta-analysis Protocols on June 17, 2020 (INPLASY202060063).

## 1 Introduction

Traumatic or non-traumatic intraventricular hemorrhage (IVH) frequently leads to post-hemorrhagic hydrocephalus (PHH) as a result of obstruction of cerebrospinal fluid (CSF) outflow, dysfunction of CSF absorption, or CSF hypersecretion.^[1,2]^ The majority of patients with PHH are shunt-dependent since conservative treatments or temporary CSF drainage always fail to divert the accumulated CSF.^[3]^ The most commonly used option is ventriculoperitoneal shunt (VPS).^[4]^ Lumboperitoneal shunt (LPS), as an alternative option, has become the first-line treatment for patients with idiopathic normal pressure hydrocephalus (INPH) in Japan.^[5]^ The indications for performing LPS have recently broadened to other types of communicating hydrocephalus, including communication PHH.^[4,6]^ Increasing number of studies reported that LPS had some advantages over VPS, including avoidance of access to lateral ventricles, minimal brain injury, and low incidence of postoperative infection.^[7]^ Although shunt implantation could effectively alleviate the symptoms and improve the neurological function, the long-term shunt failure is an unacceptably common outcome.^[8,9]^ The best treatment option for patients with PHH still remains unknown.

## 2 Objective

The objective of the current meta-analysis is to systematically evaluate the long-term outcomes of patients with PHH treated by VPS and LPS.

## 3 Methods

### 3.1 Study registration

The study is priorly registered through International Platform of Registered Systematic Review and Meta-analysis Protocols on June 17, 2020 (INPLASY202060063). This study protocol was prepared according to the guidelines of preferred reporting items for systematic review and meta-analysis protocols statement.^[10]^

### 3.2 Eligible criteria

#### 3.2.1 Types of studies

Randomized controlled trials (RCTs) and non-RCTs that simultaneously evaluated the clinical outcomes of patients with PHH treated by VPS and LPS will be included. Other studies, including retrospective studies, case reports, case series, research letters, reviews, noncomparative studies, and meeting abstracts, will be excluded.

#### 3.2.2 Interventions

##### 3.2.2.1 Study group

Patients with PHH treated by VPS.

##### 3.2.2.2 Control group

Patients with PHH treated by LPS.

##### 3.2.2.3 Participants

Adult patients (age >18 years) who were diagnosed as PHH according to medical history, clinical manifestations, brain imaging, and supplemental tests regardless of gender, racial, and region, will be included.

##### 3.2.2.4 Language

English and Chinese studies will be considered.

##### 3.2.2.5 Search strategy

We search literatures through PubMed, Web of Science, Embase, Cochrane Library, China National Knowledge Infrastructure (CNKI), the Chinese Science and Technology Periodical Database (VIP) and Wan fang databases, and Chinese Biomedical Literature Database (CBM) from its beginning to June 15, 2020. The search strategy is (“clinical outcomes” OR “efficacy” OR “safety” OR “follow-up” OR “shunting outcomes”) AND (“post hemorrhagic” OR “intracranial hemorrhage” OR “intraventricular hemorrhage” OR “subarachnoid hemorrhage”) AND (“hydrocephalus” OR “ventriculomegaly” OR “accumulation of cerebrospinal fluid”) AND (“shunts” OR “ventriculoperitoneal shunt” OR “lumboperitoneal shunt”). An example of search strategy in PubMed database is shown in Table 1.

**Table 1:**
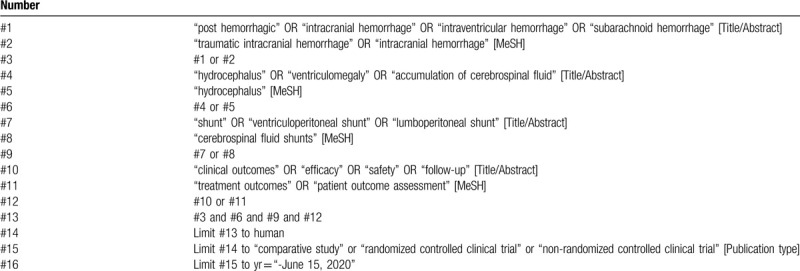
Search strategy in PubMed.

### 3.3 Outcomes

#### 3.3.1 Primary outcomes

The primary outcome is the rate of shunt failure after shunt implantation. According to the previous studies, shunt failure is defined as the occurrence of clinical or radiological signs of shunt obstruction, breakage, tubing exposure, malfunction, or infection requiring shunt revision, or shunt-related morbidity and mortality.

#### 3.3.2 Secondary outcomes

The secondary outcome is the rate of complications that are associated with shunt surgery.

### 3.4 Data collection and analysis

#### 3.4.1 Study selection

All eligible studies will be input into Endnote X7 software after 2 experienced and practiced evaluators review the tile, abstract, and full text of each study according to the inclusion and exclusion criteria. Re-evaluation by a third reviewer is required while there are different judgments on study selection. Duplicative records will also be removed. The reasons of all removed trials will be listed in a flowchart of study selection.

#### 3.4.2 Data extraction

Once the studies are included, 2 independent assessors will collect data including corresponding author, date of publication, study design, country of study, study period, participants (numbers, age, gender, race, clinical features, intervention, types of shunt system, postoperative outcomes, follow-up period), shunt outcomes, and complications. Re-evaluation by a third reviewer is required while there are any debates on data extraction.

#### 3.4.3 Dealing with missing data

We will contact the corresponding author on reasonable request if there is missing or unclear data. Finally, the studies will be removed if the data is not available despite our conscientious attempts.

#### 3.4.4 Quality assessment

The quality of included studies will be independently evaluated according to the Cochrane risk-of-bias tool containing 7 aspects. The evaluation of bias will be classified into 3 categories: high risk, low risk, and unclear risk. Re-evaluation by a third reviewer is required while there are any debates

#### 3.4.5 Publication bias

We will check the publication bias using funnel plots if at least 10 studies are included. Specifically, Begg and Egger regression is used to determine the funnel plot asymmetry.^[11]^ An independent monitoring committee, including statisticians, and data analysts, will assess the quality. We use Cochrane risk-of-bias tool to evaluate the randomized controlled trials.

### 3.5 Sensitivity analysis

The sensitivity analysis will be performed by removing 1 study each time from pooled analysis followed by reporting the results in a summary table.^[12]^

### 3.6 Data synthesis

All data are analyzed using the statistical software program Review Manager 5.3 (Nordic Cochran Centre, Copenhagen, Denmark). A 2-tailed probability values (*P*) less than .05 is considered to have statistical difference. The outcomes are mainly described as percent (%) with its 95% confidence intervals. Heterogeneity among included studies will be tested using *I*^2^ statistics. A fixed-effect model will be used if the value of *I*^2^ under 50%, and a random-effect model will be used if the value of *I*^2^ beyond 50%. The reasons of heterogeneity will be fully understood regarding the study design and patients characteristics.

## 4 Discussion

With the advent of shunt systems, CSF shunts that divert the accumulated CSF to the peritoneal cavity have long been used as the first-line treatment for patients with hydrocephalus.^[9]^ While offering some advantages over VPS, the long-term outcomes of LPS implantation, sometimes, is not as expected and the majority of patients choose VPS rather than LPS.^[13,14]^ A prospective, multicenter trial that analyze the long-term outcomes of patients with INPH treated by LPS and compare with a historical VPS control from a previous conducted trial suggested LPS presented higher risk of shunt revisions at 1 year after surgery.^[15]^ Despite of growing number of researches focusing on the benefits of LPS surgery in the treatment of INPH, no completed systematic review has compared LPS and VPS for the treatment of PHH. In this regard, our work will provide evidence for the treatment options for patients with PHH.

## Author contributions

**Conceptualization:** Junwen Guan, Chao You, Tong Sun.

**Data curation:** Yikai Yuan, Jingguo Yang, Xuepei Li

**Formal analysis:** Tong Sun, Xuepei Li

**Funding acquisition:** Chao You

**Investigation:** Yikai Yuan, Jingguo Yang, Xuepei Li

**Project administration:** Chao You

**Quality assessment:** Yikai Yuan, Yicheng Zhou

**Software:** Tong Sun, Jingguo Yang

**Supervision:** Chao You

**Validation:** Chao You

**Writing – original draft:** Tong Sun, Xuepei Li, Jingguo Yang

**Writing – review & editing:** Junwen Guan, Chao You, Xuepei Li
